# Implementation of the European health interview survey (EHIS) into the German health update (GEDA)

**DOI:** 10.1186/s13690-017-0208-6

**Published:** 2017-09-18

**Authors:** C. Lange, J.D. Finger, J. Allen, S. Born, J. Hoebel, R. Kuhnert, S. Müters, J. Thelen, P. Schmich, M. Varga, E. von der Lippe, M. Wetzstein, T. Ziese

**Affiliations:** Department of Epidemiology and Health Monitoring, Robert Koch Institute, General-Pape-Str. 62-66, D-12101 Berlin, Germany

**Keywords:** Health interview survey, Ehis, Adults, Germany, Health monitoring, Surveillance, Public health indicators, Europe

## Abstract

**Background:**

This methodological paper describes the integration of the ‘European Health Interview Survey wave 2’ (EHIS 2) into the ‘German Health Update’ 2014/2015 (GEDA 2014/2015-EHIS).

**Methods:**

GEDA 2014/2015-EHIS is a cross-sectional health survey. A two-stage stratified cluster sampling approach was used to recruit persons aged 15 years and older with permanent residence in Germany. Two different modes of data collection were used, self-administered web questionnaire and self-administered paper questionnaire. The survey instrument implemented the EHIS 2 modules on health status, health care use, health determinants and social background variables and additional national questions. Data processing was conducted according to the quality and validation rules specified by Eurostat.

**Results:**

In total, 24,824 questionnaires were completed. The response rate was 27.6%. The two-stage cluster sample method seems to have been successful in achieving a sample with high representativeness. The final micro data file was inspected, approved and certified by Eurostat. Access to micro data of the EHIS 2 can be provided by Eurostat via research contract and to the GEDA 2014/2015-EHIS public use file by the Research Data Centre of the Robert Koch Institute. First EHIS 2 results are available at the Eurostat website.

**Conclusions:**

Integrating a multinational health survey into an existing national health monitoring system was a challenge in Germany. The national survey methodology for conducting the survey had to be further developed in order to meet the overarching goal of harmonizing the health information from national statistical offices and public health research institutes across the European Union. The harmonized EHIS 2 data source will profoundly impact international public health research in the near future. The next EHIS wave 3 will be conducted around 2019.

## Background

The ‘German Health Update’ (GEDA) study is a population-based cross-sectional health interview survey conducted on behalf of the German Federal Ministry of Health by the Robert Koch Institute (RKI) in the German adult population. GEDA is one of the three components of the German Federal Health Monitoring programme at the national level being operated by the RKI [1]. The other components are the German health interview and examination surveys for children and adolescents (KiGGS) [2] and for adults (DEGS) [3]. The aim of the health monitoring programme is to provide reliable information on the population’s health status, health determinants and health care utilization. Time trends and regional differences of population health indicators can be monitored based on GEDA because of its large sample size and the regularity of the survey waves. The information obtained forms the basis for the Federal Health Reporting, the official public health statistics and epidemiological research; moreover, it serves for the planning, implementation and evaluation of public health policies. The European Health Interview Survey (EHIS) aims to provide statistical data — on a harmonized basis and with a high degree of comparability between the European Union (EU) member states — supporting the monitoring of health policies on social inclusion and protection, health inequalities and healthy ageing at the European level. EHIS data can be used as a basis for a range of purposes including national and European health monitoring and reporting, epidemiological research, construction of the European core health indicators (ECHI) [4, 5] and international comparative studies. The first EHIS wave (EHIS 1) was conducted on the basis of a gentlemen’s agreement without legal obligation. 17 EU member states participated in EHIS 1. Germany integrated parts of the EHIS questionnaire into the GEDA wave 2010. This has allowed for European comparisons for some EHIS 1-based indicators. According to Regulation 1338/2008 of the European Parliament and of the Council on Community Statistics on Public Health and Health and Safety at Work, the survey is to be conducted every five years [6]. The EHIS wave 2 (EHIS 2) was conducted in all EU member states and Iceland and Norway during the period 2013–2015 on a legally compulsory basis according to Commission Regulation (EU) No 141/2013 and its subsequent amendment to take account of the accession of Croatia to the EU (Commission Regulation (EU) No 68/2014). EHIS is part of the European Statistical System (ESS) and is operated by the national statistical offices or authorized national research institutes in the EU member states. EHIS is conducted either as a standalone survey or integrated into existing national health monitoring systems. In Germany, the latter is the case and the RKI is responsible for conducting the EHIS.

This article aims at describing the implementation of the EHIS 2 within the German Federal Health Monitoring programme. The challenges of and solutions for integrating a multinational survey into an existing national health monitoring system are discussed and further developments of the GEDA study design, data collection proceedings and contents are presented.

## Methods

### Study design and sampling procedure

Three GEDA waves have been carried out as telephone interview surveys between 2009 and 2012 in which more than 60,000 respondents participated [7]. In the current GEDA wave ‘GEDA 2014/2015-EHIS’ being described in this article, the EHIS 2 questionnaire [8] was completely integrated. According to the EHIS 2 implementing regulation [6], the reference year should be 2013, 2014 or 2015 and the data collection period should be spread over at least three months including at least one month of the autumn season (September – December). GEDA 2014/2015-EHIS was conducted between November 2014 and July 2015. The study population was defined according to regulation as persons aged 15 years and older with permanent residency in Germany. The sampling frame was changed from a random digit dialing sampling frame to a population registry based sampling frame to meet the EHIS requirements. The sampling procedure followed a two-stage stratified cluster sampling approach. In the first sampling stage, 301 communities were randomly selected as primary sampling units (PSUs) from a list of all 11,339 populated communities in Germany, stratified by 412 administrative districts and the ‘BIK region size classes’ [9] that take into account the population size as well as the regional population and employment density. The BIK classification is commonly used in Germany for regionally stratified sampling designs [10]. Sampling probabilities were proportional to the population size of the communities using the Cox procedure for controlled rounding [11]. The selection was performed by the GESIS - Leibniz-Institute for the Social Sciences in Mannheim, Germany. Sampled communities with less than 1000 residents were combined with similar small neighbor communities treated as single PSUs. Several major cities were represented by multiple PSUs due to their large populations. In the second sampling stage, individuals with a permanent residence in the sampled communities were drawn as secondary sampling units (SSUs) from the local population registers using an age-stratified random sampling procedure. Gross sample sizes for age groups were calculated according to their estimated response rates from prior pretests in order to approximate the age distribution of the population. Accordingly, age groups expected to have low response rates were oversampled in this sampling stage.

The minimum effective sample size calculated by Eurostat for the EHIS sample in Germany was *n* = 15,260 [6, 8]. This number represents the sample size that is required if the survey was based on simple random sampling with a design effect of *Deff* = 1.0. The design effect in GEDA 2014/2015-EHIS had to be presumed to be >1.0 because of the cluster effects resulting from the cluster sampling design used. Thus, a higher effective sample size had to be achieved in order to obtain survey estimates with the required precision. With a sample size of *n* = 20,000 (ca. 10,000 men and 10,000 women) and a design effect of *Deff* = 1.5, it is possible to calculate a prevalence of 20% with a 95%-confidence interval (CI) of +/− 1 percentage point for men and women according to the Wilson’s score method [12]. As a result, 67 participants were required in each of the 301 PSUs.

Another reason to aim for a higher effective sample size was the objective to perform regionally stratified analyses at the level of the 16 German federal states. The minimum sample size allocated to each federal state was *n* = 800 (ca. 400 men and 400 women). With *n* = 400 and a design effect of D*eff* = 1.5, it would be possible to estimate relatively high prevalence rates, such as a prevalence of 20%, with a 95%-CI of approximately 10 percentage points (15.5% - 25.4%) according to Wilson’s score method.

**Fig. 1:**
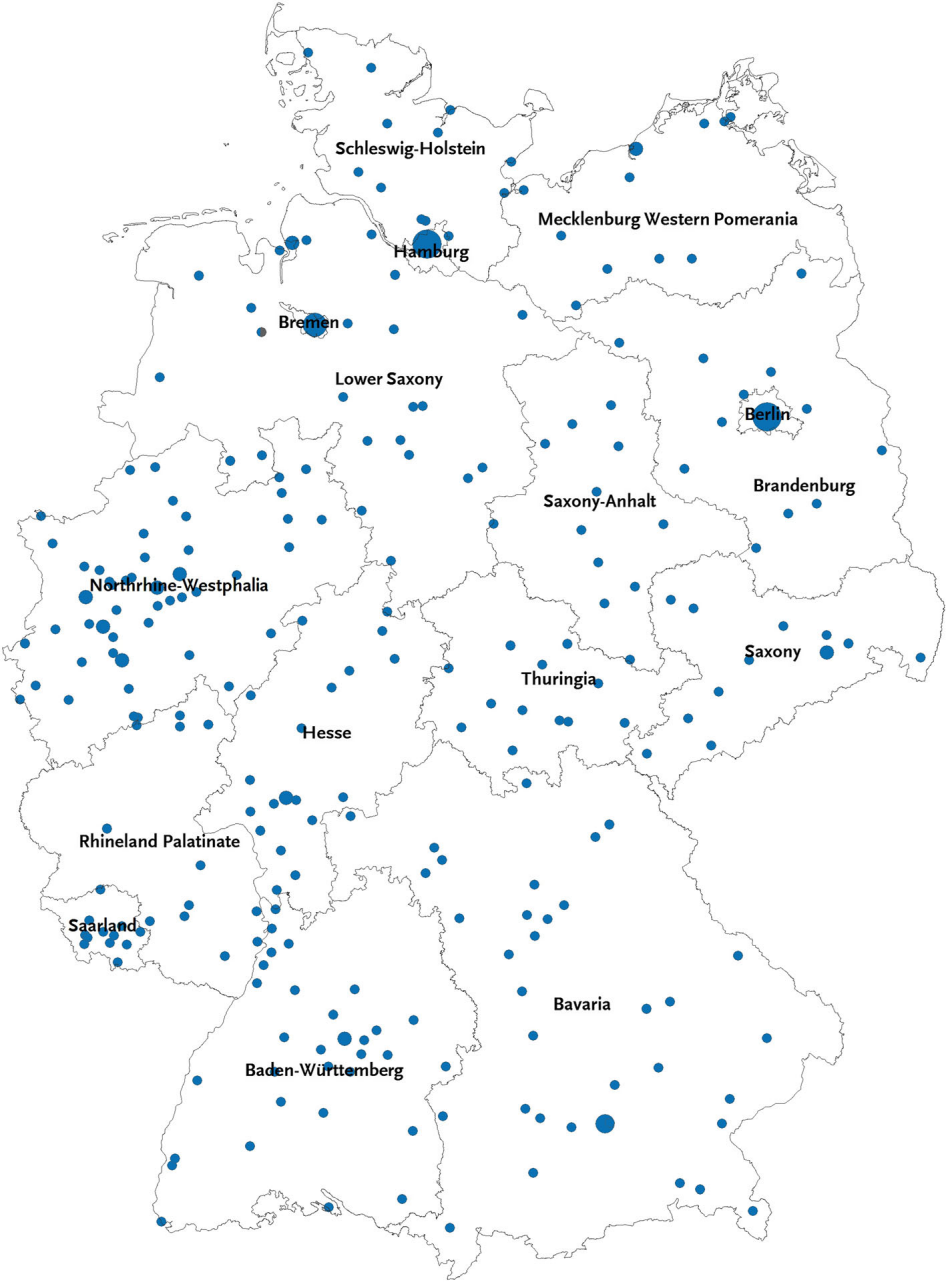
Map of GEDA 2014/15-EHIS sample points

Therefore, in less populous federal states, PSUs were oversampled to achieve a minimum number of 12 in order to ensure that a sample size of 800 participants was obtained for each state. Fig. 1 illustrates the 301 sampled communities and their location in the 16 German federal states. The size of the points presented in the figure is proportional to the number of PSUs they represent. Larger cities that are represented by multiple PSUs are depicted with larger points compared to smaller communities that are represented by only one PSU.

### Recruitment of study participants and mode of data collection

The gross sample consisted of 92,771 persons aged 15 and older. GEDA 2014/2015-EHIS data was collected during all seasons to prevent seasonal bias. The sample was randomly divided into two tranches, both being of similar size and with similar regional distribution. In the first tranche invitation letters were sent out on November 11th 2014 and survey participation was possible during autumn and winter. For the second tranche invitation letters were sent out on March 31st 2015 and data collection took place during spring and summer.

**Fig. 2:**
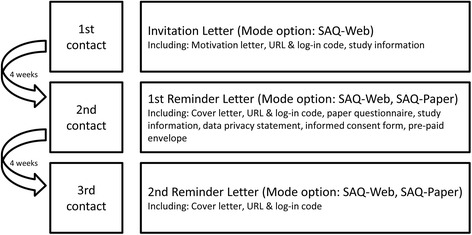
Overview of the contact process

The meaning of the term interview in the context of a ‘health interview survey’ (HIS) has a broad understanding including personal face-to-face or telephone interviews as well as data collection in a written manner by self-administered questionnaires. In GEDA 2014/2015-EHIS the latter was used. An informed sequential mixed-mode data collection design was used for GEDA 2014/2015-EHIS. The methodology was developed using the experience obtained from the methodological pilot study GEDA 2.0 [13, 14] and the special survey GEDA 2013s [15]. Mixed-mode here is defined as using one survey instrument with two or more data collection modes. In GEDA 2014/2015-EHIS, two different modes of data collection were used: a self-administered web questionnaire (SAQ-Web) and a self-administered paper questionnaire (SAQ-Paper). Fig. 2 illustrates the initiating contact process. Participants were invited in the first invitation letter to participate via SAQ-Web and informed that they will receive a SAQ-Paper if they have not participated by web mode within four weeks. The letter included a URL and a unique log-in code to access the informed consent form and the SAQ-Web online, as well as detailed information on the purpose and contents of the study and the data protection and confidentiality proceedings. The letter also offered an opportunity to refuse participation by telephone, e-mail, fax or mail. Those who had refused had been removed from the data base according to German data protection rules. A reminder letter was sent to everyone who had not completed the interview four weeks after the initial letter was sent out. The reminder letter included the SAQ-Paper, an informed consent form and a pre-paid reply envelope. The URL and log-in code were provided in this letter again to still give the opportunity to participate via SAQ-Web. Four weeks subsequent to the first reminder letter, a second reminder letter was sent to everyone who had still not responded. This letter only consisted of a cover letter and the URL and log-in code information. Addressees who were found to be ineligible were also excluded from the reminder process. This was mainly due to the fact that the named person no longer lived at the address for which he or she was sampled.

A hotline was available for all invitees throughout the 11 months of the survey period. The phone was staffed during business hours. At peak times, up to four colleagues operated the hotline simultaneously. The survey website [16] was another possibility for potential participants to get additional information on the content or the procedures of the study.

Two incentive strategies were applied in GEDA 2014/2015-EHIS. Respondents aged 15 to 34 years were guaranteed a 10 €-voucher after taking part in the survey. Respondents older than 34 years were offered participation in a lottery in which they had the chance to win one of 400 50€-vouchers. Different incentive strategies were tested in a previous feasibility study. Results showed that the effectiveness of different incentives varies depending on the age of the respondents. For participation the guaranteed 10 €-voucher had the strongest positive effect on the age group 15 to 34 years. For respondents older than 34 years the lottery option had either the same effect or in the older age groups even a stronger effect. Based on this experience and because of budget reasons in GEDA 2014/2015-EHIS the guaranteed 10 €-voucher was only offered to respondents younger than 35 years and everyone else was able to take part in the lottery.

Regional and local newspapers were contacted five weeks prior to sending out the invitation letters in order to increase awareness of the survey taking place. Especially in rural areas, local newspapers provide a good opportunity to reach a large number of residents. Newspaper editors were contacted by e-mail and were requested to publish some information about the GEDA study. The newspapers were provided with a press release summarizing the most important points about the survey. In total 90 of the 270 contacted newspapers published information about the survey around the time when the invitation letters were sent to potential participants.

The returned paper questionnaires were considered as valid interviews only when the respondents signed the written informed consent form. Was this signature missing on the consent form the person was contacted again by mail. It was assumed that the participant forgot to sign it rather than intentionally left the form blank. Out of 430 cases where participants were contacted again with an explanatory letter including the consent form and a pre-paid envelope 264 signed forms were returned. After this reminder action we had 166 questionnaires left without a signed consent. These were considered as non-interviews. Participants who chose to take part using the SAQ-Web were informed about data protection and the confidential use of their data on an introduction screen. The online survey was set up in the way so that the potential participant had to tick a box to indicate that they had read and agreed to the conditions of the study. The survey questions could not be viewed by any participant beforehand. For online participants, it was possible to pause the filling out process. The respondents could log back in using the same log-in code as before at any point in time later within the field period and could then continue from the position where they had left off.

### Instruments

The GEDA 2014–2015/EHIS questionnaire has two components. It implements the EHIS 2 in Germany and complements the EHIS questionnaire with national-level questions in order to sustain time series of GEDA as well as to get information on additional nationally relevant topics (Table 1). The GEDA data profile has been described in detail elsewhere [7].

**Table 1:** Overview of the contents of the survey instrument

Code	EHIS questions	Additional national questions
EHSM	European Health Status Module	
HS	Health Status – Minimum European Health Module	
CD	Diseases and chronic conditions; self-reported 12-month-prevalences	Diseases and chronic conditions, ever diagnosed by a medical doctor
		Additional diseases were included such as heart failure, cancer, osteoporosis, gastro-intestinal diseases, increased blood-lipids
		Module on diabetes care: age of onset, gestational diabetes, recent treatment, preventive measures, consequences of diabetes, family history etc.
AC	Accidents and injuries	Work-related accidents and injuries
AW	Absence form work (due to health problems)	Officially recognized disability
PL	Physical and sensory functional limitations	
PC	Personal care activities	
HA	Household activities	
PN	Pain	
MH	Mental health	
ECHM	European Health Care Module	
HO	Use of inpatient and day care	
AM	Use of ambulatory and home care	Number of consultations of medical doctors/specialist in the past 12 month
MD	Medicine use	
PA	Preventive services	Module on vaccinations: influenza vaccination in the last winter seasons, vaccinations against tetanus, measles, pertussis, pneumococcus, knowledge and attitudes on vaccinations etc.
		Reason for last mammography
EHDM	European Health Determinants Module	
BM	Weight and height	
PE	Physical activity / exercise	Questions on stages of change for physical activity (according to transtheoretical model [17]), motivations and barriers for physical activity
FV	Consumption of fruit and vegetables	Number of portions per week
SK	Smoking	Age of onset, age of cessation, tobacco products, current/never/former smokers
AL	Alcohol consumption	
SS	Social support	
IC	Provision of informal care or assistance	
		GEDA additional modules
		Health awareness, self-efficacy, addiction problems in the social network, health literacy [15, 19, 20], working conditions, knowledge about stroke symptoms
	Core social variables	
	Sex, age, country of residence birth, and citizenship, degree of urbanization, marital status, level of education, labour status, employment status, economic sector in employment, household composition, household income.	Working-related health risks, unemployment, subjective social status, health insurance, country of birth of mother and father

Adapted from Eurostat 2013, page 8,9 [8]

*EHIS* European Health Interview Survey, *GEDA* German Health Update

The EHIS aims at offering comprehensive data on the health status of a population and health-related topics on a representative basis. EHIS consists of four modules on health status, health care use, health determinants and social background variables. In the respective modules, the following topics are covered: Health status (self-perceived health, chronic diseases, limitation in activities, mental health, pain, accidents, etc.), health care (use of different types of health care services including hospitalizations, consultations, prevention, use of medicines but also unmet needs for health care), health determinants (smoking and alcohol consumption, body weight, physical activity, dietary habits, etc.), and background variables on demography and socio economicstatus such as sex, age, household type, education, income, employment etc.

The EHIS 2 questionnaire and its implementing guidelines can be found in the EHIS 2 methodological manual [8]. During the implementation of the EHIS 2 in Germany, all methodological guidelines were strictly followed. The EHIS 2 questionnaire was translated into German according to the recommended translation protocol [8]. For some modules, available validated German versions were used. Some linguistic adaptations were performed to meet the requirements of self-administered questionnaires.

Additional national questions, such as modules on physical activity, health literacy, subjective social status and working conditions, were added to the EHIS 2 questionnaire (Table 1). The stages of change for physical activity were assessed according to the ‘transtheoretical model’ [17] and the barriers and motivations to physical activity were assessed according to the Special Eurobarometer Survey 412 [18]. Health literacy was determined by an instrument developed within the European Health Literacy Project (HLS-EU) [19]. In GEDA 2014/2015-EHIS, the 16-item short version of this instrument (HLS-EU-Q16) [15, 20] was included in the questionnaire. Participants’ subjective social status was measured using a German version of the MacArthur Scale [21], which was originally developed by Adler et al. [22] for the United States adult population.

### Data management and quality assurance

Several quality assurance procedures were undertaken during the data processing phase of the study. Article 6 of the Commission’s implementing regulation for the EHIS 2 requests from the EU member states that finalized, validated and weighted microdata and quality-related reference metadata must be provided in accordance to the quality and validation rules specified by Eurostat. The data management process for the GEDA 2014/2015-EHIS was coordinated by the Research Data Centre at the RKI. All validation rules (skip, range and consistency checks) provided by Eurostat were strictly followed and processed. The data file was further checked with the provided ‘EDIT’ validation tool – a software designed to check whether the data set was correctly cleaned. The final micro data file was delivered to Eurostat via EDAMIS in June 2016 and was inspected, approved and certified by Eurostat. The metadata and quality reporting follows a standard template developed by Eurostat that contains information on the data file quality and can be accessed on the Eurostat website [23].

## Results

### Response rates and final study sample

In total, 24,824 questionnaires were completed; 11,253 via SAQ-Web (45.3%) and 13,571 via SAQ-Paper (54.7%). The response rates were calculated according to the standards of the American Association of Public Opinion Research (AAPOR) [24]. For detailed sample distribution, see Table 2.

**Table 2:** Full sample dispositions consistent with American Association for Public Opinion Research standards for mail surveys of specifically named persons, offering two modes of participation (web & paper) [24, 41]

	AAPOR Code	N	%
Returned Questionnaire, Interview (I)	1.0000		
Complete (I)	1.1000	24,824	27.6
Eligible, Non-Interview (R + NC + O)	2.0000		
Refusal and Breakoff (R)	2.1000		
Refusal	2.1100	321	0.4
Other Person Refusal/Household-level Refusal	2.1110	456	0.5
Known-respondent Refusal	2.1120	4012	4.5
Read receipt confirmation, Refusal (web)	2.1122	595	0.7
Breakoff/Implicit Refusal^a^	2.1200	657	0.7
Non-Contact (NC)	2.2000		
Respondent unavailable during field period	2.2500	65	0.1
Other, non-refusals (O)	2.3000		
Deceased respondent including Postal Service category: Deceased^b; c^	2.3100		
Physically or mentally unable/incompetent	2.3200	873	1.0
Language problem	2.3300	68	0.1
Someone other than designated respondent completes part/all of questionnaire	2.3600	153	0.2
Miscellaneous^d^	2.9000	166	0.2
Unknown eligibility, Non-Interview (UH + UO)	3.0000		
Nothing known about respondent or address/Unknown households (UH)	3.1000	57,669	64.1
Housing unit, unknown if eligible respondent/Unknown other (UO)	3.2000		
Postal Service category: Refused to accept^e^	3.2310	110	0.1
Unknown if person is a HH resident/mail returned undelivered^f^	3.3000		
Adjusted Gross Sample (Total sample used)		89,969	100
Not eligible (NE)	4.0000		
Out of Sample^g^	4.1000	2645	
Other	4.9000	157	
Gross sample		92,771	
Response Rate 1 I/((I) + (R + NC + O) + (UH + UO))			27.6

^a^Cut-off point for this category is less than 70% of questions are answered

^b^Corresponding Deutsche Post category used is ‘Empfänger soll verstorben sein‘

^c^Deceased cases will only be classified as part of code 2.31 if person has died after the first contact/during field period. If status occurs prior to first day of the field period, case is treated as part of code 4.90 (ineligible)

^d^Cases of questionnaires sent back without the signed consent form are classified under this category

^e^Corresponding Deutsche Post category used is ‘Annahme verweigert‘

^f^Cases will only be classified under this category if status was determined after the first contact/during field period. If status occurs prior to first day of the field period, case would be treated as part of code 4.10 (ineligible)

^g^For example: if named person no longer lives at the address for which he or she was sampled, it makes the person ineligible and s/he is out of the sample

The response rate 1 (RR 1) was 27.6%. RR1 is the number of complete interviews divided by the number of interviews plus the number of non-interviews plus all cases of unknown eligibility. It is also called the Minimum Response Rate [24]. The refusal rate (REF) was 6.7%. The REF measures the proportion of all cases in which a respondent refuses to do an interview or breaks-off an interview.

Table 3 shows the response rates stratified by sex and age groups. In general, the highest response rates are observed in the age groups 55 to 74. There are differences according to sex, with the women having higher response rates in all age groups until the age of 64. In contrast, in the age groups of 65 plus men have higher response rates than women. Furthermore, while in women there are small differences according to age (except for those older than 75), men from the younger age groups have a lower response rate than men from the older age groups.

**Table 3:** Response rates according to sex and age groups

Age group	Men		Women		Total	
	*N* responded	Response (%)	*N* responded	Response (%)	*N* responded	Response (%)
15–17	384	22.5	424	25.8	808	24.1
18–24	951	22.4	1378	34.6	2328	28.3
25–34	1323	22.6	1813	32.1	3136	27.3
35–44	1585	21.0	2164	28.7	3749	24.8
45–54	2381	26.5	2886	32.1	5267	29.3
55–64	1698	30.2	2012	33.6	3710	32.0
65–74	1683	35.2	1682	31.7	3365	33.4
75+	1252	26.7	1209	16.2	2461	20.2
15+	11,256	25.9	13,568	29.2	24,824	27.6
18+	10,872	25.3	13,144	27.5	24,016	26.9

### Representativeness and weighting

The two-stage stratified cluster sample method seems to have been successful in achieving a sample with a high representativeness. Table 4 illustrates that the achieved sample distribution comparing the crude sample with the reference population is satisfactory in relation to the sex, age and federal state distribution. The population projection of the Federal Statistical Office of 31 December 2014 [25] was used as reference population*.* The representativeness of the results can be further increased by applying weighting procedures. Two cross-sectional weighting factors are available for the GEDA 2014/2015-EHIS data file, the ‘EHIS weight’ and the ‘GEDA weight’. Table 4 presents a comparison of the proportions of characteristics when using the different weighting factors. The EHIS weight targets the total study population 15 years and older and is the product of a design weight and an adjustment weight. The design weight considers the sampling design, which was described in detail above. The adjustment weight considers the age and sex distribution as well as the structure of federal states and community and population size structure between urban and rural areas (region) according to the population projection of the Federal Statistical Office [25]. The EHIS weight should be used for international comparative data analyses at the European level. The GEDA weight (in Table 4) targets the population 18 years and older and was constructed in line with the weighting method of the previous GEDA waves and the other components (KiGGS and DEGS) of the German Health Monitoring programme at RKI. In addition to the design and adjustment weights described previously for the EHIS weight, the GEDA weight also takes into consideration the level of education. The level of education is defined in line with the International Standard Classification of Education (ISCED) 11 [26] using the education distribution of the German Microcensus 2013 [27] as the reference standard. The Microcensus is an representative annual sample survey that collects information of 1 % of the German population to obtain official statistics on the German demography and the labour market in Germany [27, 28]. The GEDA weight should be used for time trend, prevalence and cross-sectional analyses at the national level.

**Table 4:** Distribution of selected respondents characteristics according to the crude sample, the weighted samples and the reference population

	Crude sample		‘EHIS weight’^a^ (design-weights + adjustment for sex, age and region)	‘GEDA weight’^b^ (Design weights + adjustment for sex, age, region and education)	Reference population: sex, age, federal state according to population projection^c^, population structure of 31 December 2014; education according to Microcensus 2013^d^	
Characteristics	Age group 15+	Age group 18+	Age group 15+	Age group 18+	Age group 15+	Age group 18+
Sex, % (n)						
Women	54.7% (13568)	54.7% (13144)	51.0%	51.1%	51.0%	51.1%
Men	45.3% (11256)	45.3% (10872)	49.0%	48.9%	49.0%	48.9%
Age, in years						
Mean (95%CI)	49.00 (48.77–49.23)	50.11 (49.88–50.34)	48.94 (48.69–49.18)	50.12 (49.86–50.38)	n.a.	n.a.
Range	15–103	18–103	15–103	18–103	n.a.	n.a.
Age groups, % (n)						
15–17 years	3.3% (808)	n.a.	3.5%	n.a.	3.5%	n.a.
18–29 years	15.7% (3888)	16.2% (3888)	16.3%	16.9%	16.3%	16.9%
30–44 years	21.5% (5325)	22.2% (5325)	21.6%	22.2%	21.5%	22.3%
45–64 years	36.2% (8977)	37.4% (8977)	35.1%	36.4%	35.2%	36.5%
≥ 65 years	23.5% (5826)	24.3% (5826)	23.6%	24.5%	23.5%	24.3%
Education, % (n)						
Missing	0.2% (60)	0.2% (53)	0.2%	0.3%	n.a.	n.a.
ISCED 1–2	18.0% (4458)	15.5% (3723)	19.1%	19.4%	n.a.	19.6%
ISCED 3–4	49.6% (12306)	51.0% (12241)	48.6%	58.0%	n.a.	58.3%
ISCED 5–8	32.2% (8000)	33.3% (7999)	32.1%	22.2%	n.a.	22.1%
Federal state, % (n)						
Schleswig-Holstein	4.1% (1010)	4.1% (977)	3.5%	3.5%	3.5%	3.5%
Hamburg	4.2% (1045)	4.2% (1017)	2.2%	2.2%	2.2%	2.2%
Niedersachsen	8.3% (2060)	8.2% (1981)	9.6%	9.6%	9.6%	9.6%
Bremen	3.6% (894)	3.6% (876)	0.8%	0.8%	0.8%	0.8%
Nordrhein-Westfalen	16.6% (4109)	16.6% (3981)	21.7%	21.6%	21.7%	21.6%
Hessen	6.0% (1498)	6.0% (1452)	7.5%	7.5%	7.5%	7.5%
Rheinland-Pfalz	3.9% (958)	3.9% (926)	5.0%	4.9%	5.0%	4.9%
Baden-Württemberg	10.5% (2599)	10.5% (2515)	13.1%	13.1%	13.1%	13.1%
Bayern	13.2% (3280)	13.2% (3169)	15.6%	15.6%	15.6%	15.6%
Saarland	3.7% (918)	3.7% (891)	1.2%	1.2%	1.2%	1.2%
Berlin	3.8% (941)	3.8% (908)	4.3%	4.3%	4.3%	4.3%
Brandenburg	4.5% (1110)	4.5% (1070)	3.1%	3.1%	3.1%	3.1%
Mecklenburg-Vorpommern	4.2% (1046)	4.2% (1009)	2.0%	2.0%	2.0%	2.0%
Sachsen	5.0% (1251)	5.0% (1210)	5.0%	5.1%	5.0%	5.1%
Sachsen-Anhalt	3.9% (968)	3.9% (934)	2.8%	2.8%	2.8%	2.8%
Thüringen	4.6% (1137)	4.6% (1100)	2.7%	2.7%	2.7%	2.7%

*EHIS* European Health Interview Survey, *GEDA* German Health Update, *n.a* not applicable

^a^‘EHIS weight’ is constructed for analyses at the European level targeting the population aged 15+ years

^b^‘GEDA weight’ is constructed for analyses at the national level targeting the population aged 18+ years

^c^Federal Statistical Office (DESTATIS). Population projection [available from GENESIS online data base: https://www-genesis.destatis.de/genesis/online; accessed: 11 May 2017]. Wiesbaden: DESTATIS; 2015

^d^Federal Statistical Office (DESTATIS). German Microcensus 2013 [available from Research Data Centres of the Federal Statistical Office and the statistical offices of the Länder: http://www.forschungsdatenzentrum.de/en/; accessed: 4 November 2016]. Wiesbaden: DESTATIS; 2013

The distribution of the sample according to age, sex, education and region equalizes with the distribution within the population in Germany when applying the weighting procedures (Table 4). This enables the performance of representative analyses for the German population.

### Data access, types of analyses and first results

Macro data for the EHIS 2 can be downloaded free of charge for all EHIS 2 participating countries on the Eurostat website [29] and access to the micro data can be requested from Eurostat via a research contract. The dataset of the GEDA 2014/2015-EHIS can be provided by the Research Data Centre of the RKI [30]. The GEDA 2014/2015-EHIS data can be used for various types of data analyses such as prevalence, time trend or cross-sectional data analyses as well as international comparative analyses and epidemiological research. The large sample size obtained provides the opportunity to conduct analysis on a regional level and make stratifications according to sex and age.

First results of the EHIS 2 can be viewed at the Eurostat database [29]. Under the rubric ‘Population and social conditions’ and then ‘Health’, EHIS 2 results for selected health status and health determinants indicators can be downloaded for free. First EHIS 2 results on fruits and vegetable consumption and obesity were published in the Eurostat news releases in October 2016 [31, 32]. In the Eurostat online publication ‘Health in the European Union – facts and figures’ recent statistics on health in the European Union are provided [33].

First results of the GEDA2014/2015-EHIS on disease and chronic conditions and health behaviors will be published in the first and second issue of the Journal of Health Monitoring in March and June 2017.

## Discussion

This methodological paper presents the survey methodology of the GEDA 2014/2015-EHIS study, a population-based nationwide health interview survey that is a component of the German Federal Health Monitoring programme [1, 34]. For the first time, the EU-wide harmonized EHIS 2 Questionnaire was completely integrated into GEDA. Compared to the previous GEDA waves in 2009, 2011 and 2012, a range of innovations were introduced for GEDA 2014/2015-EHIS. The sampling design, data collection mode and data processing guidelines were modified partly due to the EHIS integration.

### Combining EHIS and GEDA

Integrating a multinational health interview survey into an existing national health monitoring programme has certain advantages and disadvantages. The disadvantages are that every change in the question wording and the methodology of conducting the survey between two waves of data collection might lead to interruptions of existing national time trend series. It can be expected that the methodological changes introduced have partly compromised the comparability between the GEDA 2014/2015-EHIS and previous GEDA waves. The sampling design was changed from telephone samples (GEDA 2009–12) to a population-registry sample (GEDA 2014/2015-EHIS) and the data collection mode was changed from computer-assisted telephone interview (CATI) to SAQ-Web and SAQ-Paper. The possible cuts in the national time trend series of health indicators, therefore, needs to be carefully evaluated. A previous methodological study on the possible mode differences in health interview surveys indicated that the influence of the data collection mode on prevalence estimates may be minor for some health indicators, but stronger for others [13]. Differences between the CATI mode and self-administered modes (SAQ-Paper and SAQ-Web) especially were observed for certain indicators of mental health, psychosocial factors and specific health behaviors [13]. Hence, the question of whether and how time series based on the GEDA 2009–12 telephone surveys and the GEDA 2014/2015-EHIS survey are affected by mode effects depends on the health indicator under study.

The advantages are that the utilization of existing structures and the expertise of the national health monitoring programme allows for a cost-effective implementation of the EHIS in Germany. Furthermore, the European harmonization of health questionnaires permits comparing observed prevalence and time trends in health status, health determinants and health care indicators across countries at the European level. The information obtained can be used for calculating the European Core Health Indicators (ECHI) [5], which serve as a monitoring tool to reveal differences in health status, health care and health determinants between countries. Nevertheless, any cross-national differences in health indicators observed based on EHIS data should be interpreted with caution. Although great effort was undertaken to harmonize data collection standards between the EU countries, full input harmonization was not possible. The health monitoring systems of different countries have historically developed over time in each country and the systems partly used different methods for conducting surveys. Methodological differences between countries are documented in the quality reports available on the Eurostat website [23]. Designing one common health questionnaire for such a large geographical region as the European region was a challenge. Cultural differences in regards to differing norms, health habits, and health care systems etc. made it challenging to tailor survey questions that were appropriate for different settings. We cannot exclude the possibility that national particularities compromise the comparability of results. Furthermore, differing geographical and climate conditions needs to be considered when interpreting the research findings. For example, the comparative findings of a health behavior such as bicycling for transportation need to be contextualized because it might be a common behavior in well-conditioned countries such as the Netherlands or Denmark but not so in countries with extreme weather or geo conditions where other forms of physical exercise are more common.

### Data quality, participation and representativeness

Several arrangements were undertaken to improve the data quality and to reduce information bias for the EHIS. The European Commission issued a grant for a project on the ‘improvement of the EHIS modules on alcohol consumption, physical activity and mental health’ [35]. The aim was to improve the EHIS 1 questionnaire for the EHIS 2. The EHIS workshop in Berlin in October 2010 served to evaluate the performance of the EHIS instrument in the EHIS 1 and to identify needs for revisions. In this project, the European Health Interview Survey - Physical Activity Questionnaire (EHIS-PAQ) was developed and cognitively tested and validated [36-38], the alcohol consumption module was modified [35] and the ‘Patient Health Questionnaire - 9 items’ (PHQ-9) depressive symptoms screener [39] was added to the EHIS 2 questionnaire.

Participation rates between surveys and countries differ significantly, which can be observed on the ‘European Health Interview & Health Examination Surveys (HIS/HES) Database’ which lists participation rates from health surveys conducted in Europe, the USA, Canada and Australia until 2009 [40]. Many different methods have been applied to calculate response rates [41]. This makes it difficult to compare participation rates across surveys and countries. The overall response rate of GEDA 2014/2015-EHIS was 27.6% (calculated according to AAPOR standard [24]). This magnitude is more or less in the range of the response rates of other German national panel surveys using the same response rate calculation method [42]. In Germany, like in other countries, the survey response rates have continuously declined over the last decades [42-45]. A recent review identifies factors that can increase response rates for survey questionnaires, such as providing incentives and the way the questionnaire is designed (length, layout and content) and delivered [46]. The response rate in GEDA 2014/2015-EHIS stayed in a similar range as the rates in GEDA 2009 and GEDA 2010 and was considerably higher than the rate in GEDA 2012. The change from a telephone sample to a population registry-based address sample may have contributed to this increase from the last GEDA wave, as well as incentives, which were given in GEDA 2014/2015-EHIS for the first time. As described above, the response rates were different according to sex and age groups. Keeping in mind that the young age groups received the incentive of a voucher, we can assume that the response rates could have been even lower without applying this incentive strategy. Although the response rates are the lowest in these age groups, we still obtained a sufficient number of cases. In line with the response patterns observed in GEDA, Tolonen et al. previously demonstrated, based on a time trend analysis of response rates of the Finish national health examination surveys between 1978 to 2002, that response rates have experienced a stronger decline over time in younger age groups compared to older age groups, among those who are lower educated compared to higher educated, and among women compared to men [45].

The strength of population-based national health surveys compared to other study designs is their high degree of representativeness. However, we cannot exclude the possibility that selection bias occurred at the different stages of the sampling procedure. The two weighting factors included in the data set should be used for data analyses to adjust the sample distribution to the reference standard. The EHIS weight should be used for data analyses dealing with country comparisons. The GEDA weight should be used when analyzing the data in the national context. For the purpose of national analyses that compare previous GEDA waves, only the population 18+ years should be used.

The EU-wide harmonized EHIS 2 data source will profoundly impact international public health research in the near future. The third EHIS wave (EHIS 3) will be conducted around 2019. Time trend analyses for the ECHI indicators will then become possible based on complete data, including information for all EU countries. A new implementing regulation will be legislated for the EHIS 3 using the current Framework regulation 1338/2008 as a basis. It is expected that, from the EHIS wave 4 onwards, the EHIS will be integrated into the ‘programme for social statistics’ and the legal framework regulations on ‘Integrated European Social Statistics’ (IESS) [47] .

## Conclusions

Integrating a multinational health survey into an existing national health monitoring system was a challenge in Germany as in many other EU countries. The national survey methodology for conducting the study had to be further developed in order to meet the overarching goal of harmonizing the health information from national statistical offices and public health research institutes across the EU. This process inevitably has led to an enhancement of the quality and comparability of health information in the EU.

## Abbreviations

AAPOR: American Association for Public Opinion Research
CATI: Computer-assisted telephone interview
CI: Confidence interval
Deff: Design effect
DEGS: German health interview and examination survey for adults (Studie zur Gesundheit Erwachsener in Deutschland)
ECHI: European Core Health Indicators
EHIS 1: European Health Interview Survey wave 1 2006–2009
EHIS 2: European Health Interview Survey wave 2 2013–2015
EHIS: European Health Interview Survey
EHIS-PAQ: European Health Interview Survey - physical activity questionnaire
ESS: European Statistical System
EU: European Union
GEDA: German Health Update
HIS/HES: European Health Interview & Health Examination Surveys
HLS-EU: European Health Literacy Project
IESS: Integrated European Social Statistics
ISCED: International Standard Classification of Education
KiGGS: German health interview and examination survey for children and adolescents (Kinder- und Jugendgesundheitssurvey)
PHQ-9: Patient Health Questionnaire - 9 items
PSU: Primary sampling unit
REF: Refusal rate
RKI: Robert Koch Institute
RR: Response rate
SAQ-Paper: Self-administered paper questionnaire
SAQ-Web: Self-administered web questionnaire
SSU: Sampling unit
USPS: United States Postal Service
